# How Affiliation Disclosure and Control Over User-Generated Comments Affects Consumer Health Knowledge and Behavior: A Randomized Controlled Experiment of Pharmaceutical Direct-to-Consumer Advertising on Social Media

**DOI:** 10.2196/jmir.5972

**Published:** 2016-07-19

**Authors:** David Christopher DeAndrea, Megan Ashley Vendemia

**Affiliations:** ^1^ School of Communication The Ohio State University Columbus, OH United States

**Keywords:** direct-to-consumer advertising, DTCA, social media, Facebook, pharmaceutical marketing, online promotion of prescription drugs, health communication

## Abstract

**Background:**

More people are seeking health information online than ever before and pharmaceutical companies are increasingly marketing their drugs through social media.

**Objective:**

The aim was to examine two major concerns related to online direct-to-consumer pharmaceutical advertising: (1) how disclosing an affiliation with a pharmaceutical company affects how people respond to drug information produced by both health organizations and online commenters, and (2) how knowledge that health organizations control the display of user-generated comments affects consumer health knowledge and behavior.

**Methods:**

We conducted a 2×2×2 between-subjects experiment (N=674). All participants viewed an infographic posted to Facebook by a health organization about a prescription allergy drug. Across conditions, the infographic varied in the degree to which the health organization and commenters appeared to be affiliated with a drug manufacturer, and the display of user-generated comments appeared to be controlled.

**Results:**

Affiliation disclosure statements on a health organization’s Facebook post increased perceptions of an organization-drug manufacturer connection, which reduced trust in the organization (point estimate –0.45, 95% CI –0.69 to –0.24) and other users who posted comments about the drug (point estimate –0.44, 95% CI –0.68 to –0.22). Furthermore, increased perceptions of an organization-manufacturer connection reduced the likelihood that people would recommend the drug to important others (point estimate –0.35, 95% CI –0.59 to –0.15), and share the drug post with others on Facebook (point estimate –0.37, 95% CI –0.64 to –0.16). An affiliation cue next to the commenters' names increased perceptions that the commenters were affiliated with the drug manufacturer, which reduced trust in the comments (point estimate –0.81, 95% CI –1.04 to –0.59), the organization that made the post (point estimate –0.68, 95% CI –0.90 to –0.49), the likelihood of participants recommending the drug (point estimate –0.61, 95% CI –0.82 to –0.43), and sharing the post with others on Facebook (point estimate –0.63, 95% CI –0.87 to –0.43). Cues indicating that a health organization removed user-generated comments from a post increased perceptions that the drug manufacturer influenced the display of the comments, which negatively affected trust in the comments (point estimate –0.35, 95% CI –0.53 to –0.20), the organization (point estimate –0.31, 95% CI –0.47 to –0.17), the likelihood of recommending the drug (point estimate –0.26, 95% CI –0.41 to –0.14), and the likelihood of sharing the post with others on Facebook (point estimate –0.28, 95% CI –0.45 to –0.15). (All estimates are unstandardized indirect effects and 95% bias-corrected bootstrap confidence intervals.)

**Conclusions:**

Concern over pharmaceutical companies hiding their affiliations and strategically controlling user-generated comments is well founded; these practices can greatly affect not only how viewers evaluate drug information online, but also how likely they are to propagate the information throughout their online and offline social networks.

## Introduction

The emergence of new interactive communication media has drastically affected the way many people seek out health information and discuss health topics. More than 70% of Internet users seek health information online for themselves and others; 55% of all users go online to diagnose ailments, 40% go online to seek information about medical treatments, and over 15% go online to look up drugs that they saw advertised [1]. Given the amount of people who use the Internet for health information seeking, it is not surprising that pharmaceutical companies have increasingly marketed their drugs through interactive websites and social media [2,3]; this practice is commonly referred to as direct-to-consumer advertising (DTCA). Recent estimates indicate that online pharmaceutical DTCA expenditures have continued to increase, while DTCA spending through more traditional broadcast media (eg, TV) has decreased. Due to its unparalleled scope and a complete lack of rigid law enforcement, online DTCA contributes to the characterization of Internet activity as being akin to the “Wild West” [4]. Overall, there is a critical need for more research that examines how online DTCA affects consumer health knowledge and decision making [5].

This study examines two serious concerns about online DTCA that researchers and health professionals view as essential to address [5-8]. The first major concern is that pharmaceutical companies might market their drugs on social media indirectly through seemingly neutral third-party sources that are, in reality, controlled or influenced by the pharmaceutical companies [5-7,9]. We examine the ramifications of such practices on social media for both individual commenters and for organizations that post drug information. The second major concern relates to the practice of strategically controlling user-generated contributions. For instance, companies might present “...moderated forums/sites that appear interactive but only offer one-sided communication” (p 824 [9]). Likewise, it is “...possible for manufacturers to support third-party bloggers, posters, and Twitter users who make flattering claims and discredit negative claims about their products in online discussions” (p 2088 [7]).

To understand the magnitude of these concerns, we conducted an experiment that examines how (1) disclosing an affiliation with a drug company, and (2) strategically controlling user-generated comments affects the evaluation of drug information provided on social media. Specifically, we examine the degree to which these concerns and practices affect peoples’ trust in multiple information sources, their likelihood of recommending a pharmaceutical drug to friends and family, and their likelihood of sharing pharmaceutical drug information with others in their online social network.

### Direct-to-Consumer Advertising

Although proponents argue that DTCA has benefits, such as educating consumers and improving patient-physician interaction, opponents argue that it has many harmful effects, such as misinforming patients and overemphasizing benefits [10]. The United States and New Zealand are the only two developed countries where DTCA is legal [11]. The US Food and Drug Administration (FDA) is responsible for DTCA oversight and has regulated drug marketing that appears through traditional broadcast media, banning misleading statements and creating categories for different types of advertisements. Product claim ads mention a specific drug by name and the ailment it intends to treat. This type of advertisement must follow a “fair balance” rule, meaning that benefits and risks are given equal coverage. Help-seeking ads, which mention an ailment but not the name of a drug, and reminder ads, which mention a drug but not what it treats, are not required to meet the fair balance rule.

The emergence of social media and other interactive platforms has only exacerbated concerns related to DTCA leading prominent scholars and health professionals to wonder “...whether regulatory responses by FDA are responsive and adaptive enough to address the inherent challenges faced by a universe of digital and Internet-based forms of DTCA” (p 271 [12]). Specifically, researchers and health professionals are extremely concerned that companies will market their drugs online in ways that (1) obscure the role companies play in producing drug information, and (2) strategically control user-generated contributions to promote a favorable, one-sided view of a company and its products [5,8,12]. A recent content analysis clearly documents how prominently pharmaceutical companies are using Facebook, YouTube, and Twitter for promotional activities [3]. In order to better understand how these specific concerns about online DTCA might affect the evaluation of drug information appearing on social media, we draw on research that more broadly examines how features of new media affect the evaluation of information.

### Warranting Theory

Researchers have applied warranting theory [13,14] to understand how features of new media affect evaluations of people [15], companies [16,17], products [18], and websites [19]. A central premise of warranting theory is that people trust information more or less depending on its warranting value; the warranting value of information is defined as the degree to which information is controlled or manipulated by the target it describes. The more people perceive information about a target (eg, person, organization, company) to be under the control of the target, the less they trust the information [20].

### Masking the Identity or Affiliations of an Information Source

As in the case of online DTCA, the complexities of new media can make it difficult to know the true identity of an information source or with whom the source is affiliated. Researchers have documented the many ways that online sources try to influence viewers by masking their true identities [21,22]. The prevalence of fake online reviews, commissioned or produced by the target being evaluated [23], provides a prominent example of how source obfuscation occurs online. Warranting theory predicts that information produced by a third party is more influential the less people perceive it to be under the control of the target being evaluated [13]. A recent study supported this prediction by indicating that online reviews were less impactful the more people were uncertain about the true identity of the reviewers [16].

Overall, consumers tend to trust user-generated reviews or word-of-mouth more than traditional advertisements [24,25]. The persuasive value of personal testimonials in health settings is also well understood [6], and is what makes researchers [9] so concerned about online DTCA wherein the affiliations people or companies have with pharmaceutical companies are not disclosed. Consistent with warranting theory, it is expected that people will trust favorable information about a pharmaceutical drug on social media more the less they perceive the drug manufacturer to be affiliated with third-party information sources. This expectation applies to organizations that post drug information to social media as well as people who comment on such posts. Cues that suggest a connection between organizations or commenters and a drug manufacturer should diminish the warranting value of any favorable evaluations they produce, thus (1) making the information less trustworthy, (2) making people less likely to recommend the drug, and (3) making people less likely to share the information with others in their social network.

### Strategically Controlling User-Generated Posts on Social Media

Even when information sources truly are third parties with no connection to the target they are evaluating, features of new media can still permit targets to exert control over information they produce. Notably, targets can selectively delete some user-generated content to promote a favorable view of themselves or their products. When targets can delete user-generated content, they can exert control over third-party content, not by editing or influencing the content of messages, but by curating the composition of third-party messages that appear online. As such, targets can orchestrate the false appearance that all online commenters or reviewers share the same opinion about some issue or product (eg, they all view a drug favorably). How targets strategically control the dissemination of third-party information is increasingly important to examine because of the enhanced trust people afford user-generated content [26-29]. If no cues exist to suggest that a target is controlling the dissemination of user-generated content, viewers are likely to view the user-generated content as having a high degree of warranting value [13]. However, actions or cues that suggest that a target is controlling the dissemination of user-generated content (eg, deleting comments, restricting access to content) can lower the perceived warranting value of the information, and thus its impact on viewers’ attitudes and behaviors. A recent experiment supports this prediction in an online review setting showing that positive reviews of a company led to more favorable attitudes toward the company, the more viewers believed that the company could not control or influence what reviews were displayed [16]. We argue that the same principle should apply to control over user-generated drug evaluations on social media. Specifically, cues that indicate that user-generated evaluations of a drug have been removed from a post should increase perceptions that the drug manufacturer is controlling the dissemination of the user-generated evaluations, thus (1) lowering trust in any remaining favorable user-generated evaluations, (2) making people less likely to recommend the drug, and (3) making people less likely to share the information with others in their social network.

## Methods

### Research Design Overview

Participants in this 2 (organization affiliation) × 2 (commenter affiliation) × 2 (comment deletion) between-subjects experiment were randomly assigned to one of eight conditions. Across all conditions, participants viewed an infographic posted on Facebook by a fictitious health organization about a fictitious prescription allergy drug called OpenAir; the post was always accompanied by user-generated comments. After viewing the stimulus materials, participants completed an online questionnaire and were thanked for their participation.

### Ethics

The study was determined category 2 exempt research by the Ohio State University Institutional Review Board in accordance with the U.S. Department of Health & Human Services. It is most appropriately categorized as research that uses common survey procedures. Authors did not register the trial prospectively because they did not regard this as a clinical trial.

### Sample

The sample consisted of 674 participants from an online panel who received financial compensation in exchange for their participation. Participants ranged in age from 18 to 85 years (mean 52.86, SD 15.00) and identified as Caucasian (n=587), African American (n=44), Asian or Asian American (n=20), Native American (n=3), Hispanic (n=10), and other (n=10). More participants identified as female (n=515) than male (n=159). Participants were recruited by Qualtrics; incentives were handled by Qualtrics and disclosed to participants prior to their participation.

### Stimulus Materials

Across all conditions, participants viewed an infographic that the health organization Expert Opinions in Medicine (EOIM) posted to Facebook. The infographic always contained a quote from a medical doctor about a prescription allergy drug called OpenAir made by Darby Pharmaceuticals. The quote indicated that the drug is effective at treating seasonal allergies. The infographic was always accompanied by three comments from users who indicated that the drug is very effective. The health organization EOIM, the company Darby Pharmaceuticals, and the prescription drug OpenAir were all fictitious and were created to resemble actual entities.

For the organization affiliation factor, changes were made to the infographic to induce variability in participants’ awareness of EOIM’s affiliation with Darby Pharmaceuticals—the ostensible manufacturer of the drug OpenAir. In the nonaffiliated condition, the following statement appeared at the bottom of the infographic: “Expert Opinions in Medicine—An Independent Research Organization.” In the affiliated condition, the statement read “Expert Opinions in Medicine—A Research Organization Funded by Darby Pharmaceuticals.” In addition to this difference, a medical doctor who works for EOIM is quoted in the infographic. The nonaffiliated condition indicated the medical doctor is the Executive Director at EOIM, whereas the affiliated condition adds that he is also an OpenAir Senior Research Scientist at Darby Pharmaceuticals.

**Figure 1:**
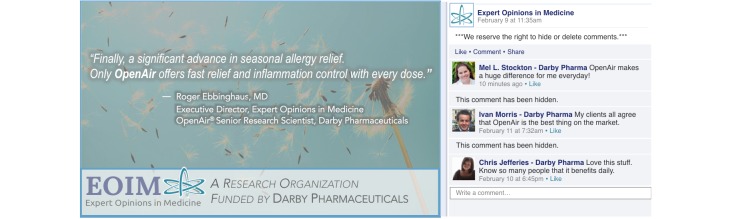
Sample stimulus Facebook infographic post: organization affiliation, comment deletion, and commenter affiliation condition.

**Figure 2:**
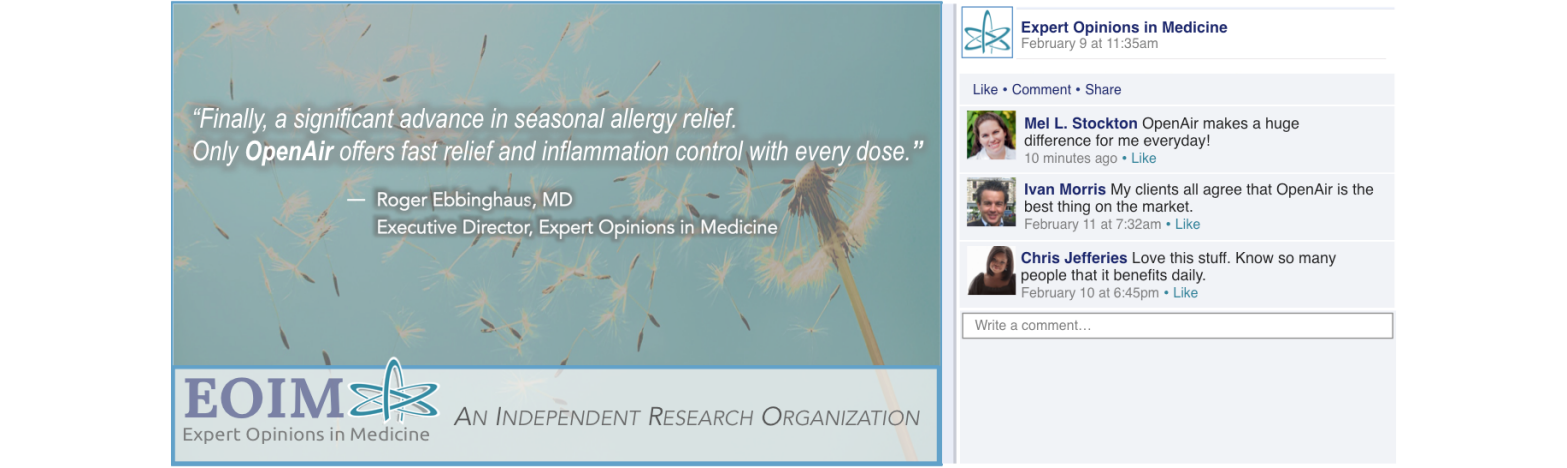
Sample stimulus Facebook infographic post: no organization affiliation, no comment deletion, and no commenter affiliation condition.

We manipulated the other two experimental factors in the comment section that accompanied the infographic. Across all conditions, there were three positive comments on the infographic post. For the commenter affiliation induction, “Darby Pharma” appeared next to the commenters’ names in the affiliation condition (eg, Mel L, Stockton-Darby Pharma). In the nonaffiliation condition, no indication was provided that the commenters were associated with Darby Pharmaceuticals. For the dissemination control induction, the caption “We reserve the right to hide or delete comments” appeared above the comments in the deletion condition. In addition, the comment section also indicated that some comments had been hidden. In the nonremoval condition, no statement was provided about the organization’s deletion policy and no cues indicated that deletion had occurred. Sample stimuli are provided in Figures 1 and 2.

### Measures

#### Organization Affiliation

The extent to which the organization EOIM was perceived to be affiliated with Darby Pharmaceuticals was assessed using three items measured on 7-point scales with endpoints ranging from extremely unlikely to extremely likely. The items were “Expert Opinions in Medicine is affiliated with Darby Pharmaceuticals,” “Expert Opinions in Medicine is connected with Darby Pharmaceuticals,” and “Expert Opinions in Medicine is funded by Darby Pharmaceuticals.” The reliability of all scales was assessed via Cronbach's alpha (α=.94).

#### Dissemination Control

The extent to which Darby Pharmaceuticals was perceived to control what comments appeared on the Facebook post was assessed with items validated in previous work [30]. Four items measured on 7-point scales were used with endpoints ranging from extremely unlikely to extremely likely (α=.97). Items included “Darby Pharmaceuticals controlled what comments appeared on the Facebook post” and “Only comments approved by Darby Pharmaceuticals appeared on the Facebook post.”

#### Commenter Affiliation

The extent to which commenters were perceived to be affiliated with Darby Pharmaceuticals was assessed using three items measured on 7-point scales with endpoints ranging from extremely unlikely to extremely likely (α=.96). Items included “The people posting comments are affiliated with Darby Pharmaceuticals” and “The people posting comments are employed by Darby Pharmaceuticals.”

#### Organization Trust

Perceived trust in the organization EOIM was assessed via four items on 7-point semantic differential scales. The stem stated “The organization Expert Opinions in Medicine is...” and the bipolar adjectives were as follows: not credible/credible, untrustworthy/trustworthy, not believable/believable, biased/ unbiased (α=.96).

#### Comment Trust

Perceived trust in the comments was assessed via five items on 7-point semantic differential scales. The stem stated “The comments people posted are...” and the bipolar adjectives were as follows: untrustworthy/trustworthy, biased/unbiased, not credible/credible, not reliable/reliable, not believable/believable (α=.97).

#### Drug Recommendation

To assess interpersonal influence, we measured the extent to which people would recommend the drug OpenAir to important others using four items measured on 7-point scales with endpoints ranging from strongly disagree to strongly agree (α=.97). Items included “I would recommend OpenAir to a friend looking for a good allergy medication” and “I would recommend OpenAir to a family member looking for a good allergy medication.”

#### Facebook Endorsement

To assess influence through mass communication, we measured the extent to which people would endorse and share the post about OpenAir with an entire online social network using four items measured on 7-point scales with endpoints ranging from strongly disagree to strongly agree (α=.98). Items included “I would ‘share’ the post by Expert Opinions in Medicine with my Facebook friends,” “I would ‘share’ the post by Expert Opinions in Medicine with my Facebook friends who have allergy problems,” “I would ‘like’ the post by Expert Opinions in Medicine on Facebook,” and “I would ‘like’ the organization Expert Opinions in Medicine on Facebook.”

#### Demographics

Whether participants suffer from seasonal allergies was assessed with one item. Participants were asked to indicate how much they agree with the following statement: “Seasonal allergies are a problem for me.” Participants were also asked to indicate whether they use Facebook. Facebook users then indicated approximately how often they access Facebook. In addition, participants responded to demographic items including gender, race/ethnicity, and age.

#### Attention Checks

In all conditions, participants were asked one question for each induction to determine the degree to which they attended to the information in their assigned condition. To check the organization affiliation induction, we asked, “According to the Facebook post you viewed, which of the following statements is true?” The answer options were, “Expert Opinions in Medicine is an independent research organization,” “Expert Opinions in Medicine is an organization that is funded by Darby Pharmaceuticals,” and “I am not sure.” To check the commenter affiliation induction, we asked, “Was there any evidence that the people who commented on the Facebook post were associated with Darby Pharmaceuticals?” and to check the comment deletion induction we asked, “Was there any evidence on the Facebook post you viewed that the organization hides or deletes comments?” The answer options for both questions were yes, no, and I am not sure.

## Results

### Data Exclusion

Before conducting the primary analyses, we examined how participants responded to the attention check questions. Participants who answered incorrectly to one or more of the attention check items were removed from the analyses (n=265). Participants who did not answer any of the questions incorrectly were retained (n=409). These two groups of participants did not significantly differ in their age, gender, ethnicity, Facebook use, or whether they suffered from seasonal allergies (all *P* values >.21).

### Analysis Plan

For each experimental factor, we first provide a *t* test that directly estimated how each induction affected perceptions of the mediating construct it was expected to vary (ie, organization affiliation, dissemination control, or commenter affiliation). Next, the macro PROCESS [31] was used to estimate the indirect effect each induction had on the outcome measures, through the proposed mediator. We first provide unadjusted indirect effect estimates and then provide covariate-adjusted estimates. A zero-order correlation matrix is provided that includes means and standard deviations for all variables in the analyses (Table 1).

**Table 1:** Zero-order correlations, means, and standard deviations (N=409).

Variable	Pearson *r*						Mean (SD)
	1	2	3	4	5	6	7		
1. Organization affiliation	1	.41**	.39**	–.31**	–.26**	–.21**	–.16*	5.44 (1.59)
2. Dissemination control		1	.57**	–.48**	–.53**	–.41**	–.40**	5.40 (1.74)
3. Commenter affiliation			1	–.42**	–.50**	–.38**	–.33**	4.74 (1.76)
4. Organization trust				1	.80**	.69**	.66**	3.70 (1.58)
5. Comment trust					1	.68**	.65**	3.55 (1.58)
6. Drug Recommendation likelihood						1	.78**	4.07 (2.15)
7. Facebook endorsement							1	2.86 (1.60)

* *P*<.01, ** *P*<.001

### Organization Affiliation

An independent samples *t* test indicated that participants perceived the organization EOIM as more affiliated with Darby Pharmaceuticals when the infographic provided a funding disclosure relative to when it did not (*t*_407_=12.23, *P*<.001, η^2^=.27). Using Model 4 of the macro PROCESS, we estimated the indirect effect the induction had through perceptions of organizational affiliation on each outcome measure; each estimate is provided with its corresponding 95% bias-corrected bootstrap confidence interval based on 10,000 resamples. Significant indirect effects were found on organization trust (point estimate –0.45, 95% CI –0.69 to –0.24), comment trust (point estimate –0.44, 95% CI –0.68 to –0.22), drug recommendation likelihood (point estimate –0.35, 95% CI –0.59 to –0.15), and Facebook endorsement likelihood (point estimate –0.37, 95% CI –0.64 to –0.16). The preceding analyses were reran controlling for perceptions of dissemination control (ie, Darby Pharmaceuticals controlled what comments appeared) and perceptions that the commenters were affiliated with Darby Pharmaceuticals. When including the covariates in the mediation models, all of the indirect effects became nonsignificant.

To further probe the nature of the relationships, we conducted tests of moderated mediation using Model 14 in PROCESS. The significant indirect effects reported previously were not moderated by participants’ perceptions that the commenters were affiliated with Darby Pharmaceuticals. However, perceptions of dissemination control did moderate three of the significant indirect effects. The overall pattern indicated that—when perceptions of dissemination control were low—the organizational affiliation induction did not indirectly affect (1) trust in the organization, (2) trust in the comments, and (3) the likelihood of recommending the drug. However, as perceptions of dissemination control increased, the size of the indirect effects significantly increased to a substantive degree. For each outcome measure, indirect effects were estimated at three levels of the moderating variable (ie, dissemination control): 1 SD below the mean, at the mean, and 1 SD above the mean. The point estimates were as follows for each outcome: organization trust (point estimates –0.09, –0.29, –0.47), comment trust (point estimates –0.02, –0.12, –0.26), and drug recommendation likelihood (point estimates 0.01, –0.14, –0.29). The direct test of moderated mediation for each outcome was as follows: organization trust index (point estimate –0.11, 95% CI –0.21 to –0.02), comment trust index (point estimate –0.09, 95% CI –0.18 to –0.002), and drug recommendation likelihood index (point estimate –0.09, 95% CI –0.18 to –0.004). A similar pattern was found for the Facebook endorsement outcome but the 95% confidence interval narrowly included zero: index (point estimate –0.08, 95% CI –0.19 to 0.01).

### Dissemination Control

An independent samples *t* test indicated that participants perceived Darby Pharmaceuticals to have more control over what comments appeared on the EOIM post when cues indicated that deletion had occurred (*t*_407_=4.39, *P*<.001, η^2^=.05). Using Model 4 of the macro PROCESS, we estimated the indirect effect the induction had through perceptions of dissemination control on each outcome measure. Significant indirect effects were found on organization trust (point estimate –0.31, 95% CI –0.47 to –0.17), comment trust (point estimate –0.35, 95% CI –0.53 to –0.20), drug recommendation likelihood (point estimate –0.26, 95% CI –0.41 to –0.14), and Facebook endorsement likelihood (point estimate –0.28, 95% CI –0.45 to –0.15). The preceding analyses were reran controlling for perceptions that the organization and the commenters were affiliated with Darby Pharmaceuticals. When including the covariates, all the indirect effects remained significant; the confidence interval of each adjusted estimate overlapped with its respective nonadjusted confidence interval. That is, the estimates did not significantly differ in magnitude.

### Commenter Affiliation

An independent samples *t* test indicated that participants perceived the commenters to be more affiliated with Darby Pharmaceuticals when the cue “Darby Pharma” appeared next to their names (*t*_407_=10.59, *P*<.001, η^2^=.22). Using Model 4 of the macro PROCESS, we estimated the indirect effect the induction had through perceptions of commenter affiliation on each outcome measure. Significant indirect effects were found on organization trust (point estimate –0.68, 95% CI –0.90 to –0.49), comment trust (point estimate –0.81, 95% CI –1.04 to –0.59), drug recommendation likelihood (point estimate –0.61, 95% CI –0.82 to –0.43), and Facebook endorsement likelihood (point estimate –0.63, 95% CI –0.87 to –0.43). The preceding analyses were reran controlling for perceptions that the organization was affiliated with Darby Pharmaceuticals and perceptions that Darby Pharmaceuticals controlled the dissemination of the comments. When including the covariates, all the indirect effects remained significant, the confidence interval of each adjusted estimate always overlapped with its respective nonadjusted confidence interval except in one case. The covariate-adjusted indirect effect on comment trust was significantly attenuated (point estimate –0.37, 95% CI –0.54 to –0.23).

### Full Sample

As noted previously, participants were removed from the analyses if they incorrectly responded to one of the attention check items. The purpose of removing the participants was to reduce error and provide a clearer test of the hypothesized relationships. However, in real-world settings, people may only provide fleeting attention to social media posts and/or may be unable to accurately recall what they viewed. As such, there is some value in being exhaustive and looking at the estimates for the full sample—even if this includes participants who made no honest attempt to read or respond to the survey items. As indicated in Table 2, all the indirect effects were significant for the full sample. Although the estimates for the full sample are attenuated relative to the reduced sample, they cannot be statistically differentiated because their respective 95% confidence intervals overlap.

**Table 2:** Indirect effects full sample (N=672): point estimates and 95% confidence intervals.^a^

Factor	Dependent variables, point estimate (95% CI)			
	Organization trust	Comment trust	Drug recommendation likelihood	Facebook endorsement
Organization affiliation	–0.32 (–0.46, –0.19)	–0.26 (–0.40, –0.13)	–0.18 (–0.31, –0.06)	–0.16 (–0.30, –0.03)
Comment deletion	–0.18 (–0.30, –0.06)	–0.21 (–0.34, –0.07)	–0.16 (–0.27, –0.06)	–0.17 (–0.29, –0.06)
Commenter affiliation	–0.47 (–0.62, –0.34)	–0.56 (–0.73, –0.42)	–0.42 (–0.56, –0.30)	–0.40 (–0.55, –0.28)

^a^ Estimates are provided with their respective 95% bias-corrected bootstrap confidence interval based on 10,000 resamples.

## Discussion

### Principal Findings

The findings from this study illustrate how important it is to better understand the effects of DTCA in a new media environment. A major concern expressed in past research [5,7,9] is that information sources might be “blurred” online, making it difficult to know when a pharmaceutical company is sponsoring or influencing the production of drug information. The results of this study suggest that cues that disclose connections between health organizations and pharmaceutical companies affect how people process drug information posted on social media. Specifically, disclosing an affiliation decreased (1) trust in an organization that posted information about a drug, (2) trust in comments posted by other site users about the drug, (3) the likelihood of recommending the drug to family or friends, and (4) the likelihood of propagating the drug message further throughout their online social network. Illustrating the complexity of new media environments that contain multiple information sources, the results also indicate that these effects are increasingly pronounced when it appears that a website proprietor controls the dissemination of user-generated comments on a webpage.

Beyond moderating the effect perceptions of organizational affiliation had on the outcome measures, perceptions of control over the dissemination of user-generated content independently affected the outcomes. This type of strategic control over user-generated content has been emphasized as a major concern for online DTCA [7,9]. The findings validate these concerns and help estimate how greatly controlling the dissemination of user-generated content can affect people who view health information posted online. Cues that indicated that an organization removed some of the user-generated comments that accompanied their posts increased people’s perceptions that the drug manufacturer was behind the removal. Notably, the more people thought that the drug manufacturer was controlling the dissemination of the user-generated comments, the less people trusted the user comments and the health organization that posted the infographic. Again, the complexities of a new media environment are illustrated. How information posted by website users is perceived to be controlled not only affects how people evaluate remaining user contributions, it also affects how people view the proprietor of the website (eg, the health organization EOIM). In addition to influencing how people trusted the user-generated comments and the health organization EOIM, control over the dissemination of user-generated content affected the likelihood that people would recommend the drug to others, and endorse/share the information with others in their online social network.

The commenter affiliation induction had similar, yet independent, effects on all the outcome measures. The results indicate that a single affiliation cue next to commenters’ names can significantly increase people’s knowledge that the commenters are affiliated with the drug company, which, in turn, can affect trust in a health organization, comment trust, drug recommendation likelihood, and the likelihood of endorsing and sharing the information with others online. Because personal testimonials from average citizens are highly influential, paid representatives or company employees who post information online without disclosing their connection to a pharmaceutical company is thought to be exceedingly troublesome [9]. The findings from this study distinctly illustrate how impactful it can be when company-affiliated individuals masquerade as neutral, third-party contributors online.

From a practical perspective, the results highlight the need for future FDA guidelines to mandate that pharmaceutical companies clearly disclose connections within messages posted to any website or social media platform that they directly fund, control, or support in some manner. Past research suggests companies have minimized or obscured such disclosures [32]. The bigger challenge, however, is whether it is feasible to regulate how pharmaceutical companies control information about their products across new media platforms that they might only indirectly influence or control [7,12]. Obviously pharmaceutical companies should not be expected to police the entire Internet. Nevertheless, regulations that only pertain to content posted to “official” company media might overlook relationships that—according to the results of this study—would greatly influence how people evaluate drug information.

The FDA has issued draft guidance documents that outline (1) when companies are responsible for user-generated content, (2) recommendations for how they should respond to user-generated content for which they are not required to respond, and (3) recommendations for how to convey risk information when online platforms have space or characters limitations [33,34]. Notably, the documents do not directly discuss how companies should respond to online platforms that make it difficult for content contributors to clearly disclose company affiliations and the precise nature of any affiliations. Ironically, the guidance document on user-generated contributions only provides guidance for handling user-generated content that comes from independent, nonaffiliated sources and exists on platforms in which companies have not edited or removed any third-party content. “A firm is thus responsible for communications on the Internet and Internet-based platforms, such as social media, made by its employees or any agents acting on behalf of the firm to promote the firm’s product, and these communications must comply with any applicable regulatory requirements” (pp 3-4 [33]). It is helpful that the guidance document confirms that existing regulations apply to content produced by company-affiliated sources and content that exists on platforms over which companies exert control. However, the problem remains that features of many prominent social media sites, such as Facebook, make it easy to mask the identity of an information source and difficult to ascertain whether companies are removing user-generated contributions.

### Theoretical Implications

The findings of this study help extend the explanatory and predictive power of warranting theory by demonstrating how the core theoretical propositions accurately explain and predict phenomena in a new context. Unlike previous tests of warranting theory that have directly examined uncertainty about the true identity of an information source online [16], this study more directly varied and measured the degree to which information sources were affiliated with the target being evaluated. Although the distinction may appear to be minor, uncertainty about the true identity of a third-party source might affect perceptions of warranting value differently relative to perceptions that a third-party source is affiliated with the target of an evaluation. For instance, an unknown source could actually be the target, someone affiliated with the target, or someone unaffiliated with the target. Future research might seek to further explore how these two considerations about the identity of a third-party source relate to one another and affect evaluations of information appearing online.

A notable finding from this work that has novel theoretical implications is that perceptions of organizational affiliation affected the outcomes differently than perceptions of commenter affiliation. It is possible that the differential effects might be attributable to how the constructs were operationalized in this study. However, it is also possible that perceived affiliations between individuals and targets might function differently than perceived affiliations between organizations/companies and targets. If the ceiling for trusting an online commenter is greater than the ceiling for trusting an organization, differential effects might be expected. Future research might seek to explore these possibilities more directly.

### Limitations

Limitations common to experimental research apply to this study. Although the results support theoretically predicted relationships, future research might seek to further the generalizability of the findings. For instance, researchers can seek to examine the effects of online DTCA across different populations, with different drug messages, and on different social media platforms. Researchers might also seek to examine how consumers’ general skepticism toward pharmaceutical marketing can moderate the effects found in this study.

A limitation specific to this study is that the attention check questions may have been overly sensitive. For instance, some participants viewed an infographic that indicated that EOIM was “An Independent Research Organization.” Participants who indicated that EOIM was not an independent organization and instead was funded by Darby Pharmaceuticals were removed. However, in some of the EOIM nonaffiliation conditions, comments were removed and commenters were affiliated with Darby Pharmaceuticals. It is possible that participants interpreted this combination of cues as indicating EOIM was not really independent, despite the claim that was made. We provide results for the full sample to overcome this limitation, but future researchers should consider how multiple cues might operate in conjunction when seeking to include attention check items designed to reduce measurement error.

### Conclusions

Pharmaceutical companies will seek to market their drugs through whatever media people regularly consume. In the current media landscape, this means drug marketing will occur through social media and online platforms that are interactive and include information from multiple sources. Any attempt to regulate online DTCA needs to thoroughly consider the unique affordances and characteristics of emerging communication technology. Whether regulations can keep pace with advances in communication technology remains to be seen. However, the results of this study provide clear evidence that obscuring (1) the true identity of an information source, (2) the affiliations of an information source, and (3) control over user-generated content can greatly influence consumer health knowledge and behavior.

## Abbreviations

DTCA: direct-to-consumer advertising
EOIM: Expert Opinions in Medicine
FDA: Food and Drug Administration
